# The Effects of a Prebiotic Formula Promoting *Akkermansia muciniphila* (AKK) on Gut Health: A Single-Centre, Randomised Controlled Trial

**DOI:** 10.7150/ijms.125881

**Published:** 2026-04-23

**Authors:** Chih-Kuan Wu, I-Shiung Cheng, Yu-Chun Chung, Megan F. Liu, Yung-Kai Lin, Yung-Hsiang Lin, Kristin Morris, Di Chang, Chi-Fu Chiang, Ming-Ta Yang

**Affiliations:** 1 Department of Urology, Taipei Medical University Hospital, Taipei, Taiwan.; 2 TMU Research Center of Urology and Kidney, Taipei Medical University, Taipei, Taiwan.; 3 Department of Physical Education, National Taichung University of Education, Taichung City, Taiwan.; 4 Center for General Education, Taipei Medical University, Taipei, Taiwan.; 5 School of Gerontology and Long-Term Care, College of Nursing, Taipei Medical University, Taipei, Taiwan.; 6 Institute of Food Safety and Risk Management, National Taiwan Ocean University, Keelung, Taiwan.; 7 Department of Food Science, National Taiwan Ocean University, Keelung, Taiwan.; 8 Graduate Institute of Biomedical Engineering, National Chung Hsing University, Taichung, Taiwan.; 9 Research & Design Center, TCI Co., Ltd., Taipei, Taiwan.; 10 Amway Innovation and Science, Ada, MI, USA.; 11 Clinical Research Center, Taipei Medical University Hospital, Taipei, Taiwan.

**Keywords:** *Akkermansia muciniphila*, gut microbiota, prebiotic supplementation, randomized controlled trial

## Abstract

**Background:**

Gut health is closely associated with metabolic homeostasis, and alterations in gut microbiota composition have been linked to inflammation and metabolic disorders. Prebiotics targeting specific taxa, such as *Akkermansia muciniphila*, have attracted interest for their potential to modulate gut microbiota composition. While preclinical studies have suggested a role for *A. muciniphila* in gut-related metabolic pathways, clinical evidence supporting its effects on microbiome regulation and metabolic outcomes remains limited. Therefore, this study aimed to explore the effects of a prebiotic blend designed to promote *A. muciniphila* on gut microbiota composition and selected physiological parameters.

**Methods:**

Seventy participants were randomized to receive either placebo or the AKK formula for 8 weeks. Anthropometric assessments, blood tests, and stool examinations were performed at baseline and at weeks 4 and 8. The main analyses were conducted in the per-protocol population (n = 25 per group).

**Results:**

The results revealed no significant markers of liver or kidney dysfunction in either group. The AKK formula group showed an increased qPCR-derived relative abundance of *A. muciniphila* compared with total bacteria. Gut microbiome analysis further demonstrated selective changes in gut microbiota composition in the AKK formula group, including an increased relative abundance of *Bifidobacterium* and decreased relative abundances of *Proteobacteria*, *Erysipelotrichia*, and *Escherichia-Shigella*. A trend toward lower gastrointestinal discomfort scores was observed in the AKK formula group during the intervention period.

**Conclusions:**

The AKK formula increased the relative abundance of *A. muciniphila*, was associated with selective modulation of gut microbiota composition, and showed a trend toward reduced gastrointestinal discomfort, supporting its potential relevance in future gut health research.

## Introduction

Gut health is essential to maintaining metabolic balance and is intricately linked to various metabolic disorders and chronic diseases. Central to this connection is the gut microbiota, where imbalances, referred to as gut dysbiosis, can promote the proliferation of pathogenic bacteria, reducing beneficial microbes and exacerbating inflammation and metabolic dysfunction 1. In recent years, probiotic therapy has gained significant attention as a potential strategy for restoring microbial balance, enhancing gut barrier integrity, and modulating immune responses. Probiotics, such as *Bifidobacterium*, *Lactobacillus*, *Lactococcus*, and *Streptococcus*, have been studied for their capacity to support gut health 2. Among these, *Akkermansia muciniphila* has emerged as a particularly promising candidate for promoting a balanced and resilient gut microbiome 3.

*A. muciniphila* is a Gram-negative, anaerobic bacterium characterized by its oval-shaped morphology and unique ability to degrade mucin, a major component of the protective mucus layer in the gut 4. This bacterium is crucial in maintaining intestinal barrier integrity and has been associated with various metabolic benefits 5. Studies indicated that *A. muciniphila* positively impacted the gut microbiota by increasing short-chain fatty acids, such as butyrate, which played a role in anti-inflammatory processes and gut barrier protection. This bacterium thrived on mucin, a component of the gut's mucus layer, reinforcing the barrier that kept harmful bacteria and toxins out of circulation. Moreover, *A. muciniphila* was associated with improved metabolic markers and was believed to help maintain a healthy weight, support immune modulation, and promote glucose regulation. Metagenomic analyses of human fecal samples have consistently shown reduced levels of *A. muciniphila* in obese individuals compared to their lean counterparts 4. *A. muciniphila* has demonstrated a significant role in supporting gut health, particularly by modulating the gut-brain axis and contributing to energy homeostasis 3. Its impact on maintaining the integrity of the gut barrier and enhancing microbial balance has drawn attention to its potential in therapeutic strategies for promoting overall gut well-being.

Recent studies have highlighted the effectiveness of certain plant extracts in promoting the growth of *A. muciniphila*, offering promising methods for gut microbiome support. The therapeutic impact of plant polyphenolic compounds such as grape seed extract has been extensively explored and applied 6. These polyphenols exert antioxidant and anti-inflammatory effects that not only help maintain intestinal stability but also directly promote *A. muciniphila* growth 7. The study indicated that proanthocyanidins from grape seed extract and catechins from green tea significantly increased *A. muciniphila* levels, fostering a balanced gut microbiome and strengthening the gut barrier. These compounds enhanced microbial diversity and gut integrity, promoting overall gut health and resilience 8. Some plant extracts are rich in prebiotics, such as inulin and fructo-oligosaccharides, which serve as nutritional substrates for *A. muciniphila* in the gut, promoting its growth and proliferation 9. Prebiotics enhance beneficial bacteria' survival rate and activity, contributing to maintaining a balanced gut microbiota 10.

The prebiotic formulation used in this study, hereafter termed the AKK formula, was designed to promote the growth of *A. muciniphila* and was composed of fermented grape powder, fructooligosaccharides, and lactitol in a proprietary ratio (w/w) provided by TCI Co., Ltd. This study aimed to evaluate the effects of this prebiotic blend on gut microbiota modulation and metabolic outcomes. Seventy participants were randomized to the placebo or AKK formula groups and were followed for 8 weeks, with assessments including body composition, blood tests, and stool examinations at baseline, week 4, and week 8.

## Methods

### Clinical trial design

This study is a double-blind trial using random allocation and a placebo-controlled design. The clinical study was approved by the Taipei Medical University Institutional Review Board (N202310027), and was registered on ClinicalTrials.gov Identifier: NCT06132269. This clinical trial period was from 2023/10/01 to 2024/09/30. Block randomization (1:1) was used to assign participants to receive either the prebiotic formula or the placebo. The randomization sequence was computer-generated, and participants were numbered sequentially upon enrollment. The allocation list was prepared and safeguarded by the principal investigator (PI) in a secure file inaccessible to recruitment staff or outcome assessors. Allocation concealment was ensured by dispensing study products according to sequentially coded kits. The investigational product and placebo were identical in appearance, volume, flavor, and packaging, and were labeled with anonymized kit codes to maintain double blinding throughout the study.

### Participants

Inclusion criteria included: (1) healthy adult males and females aged 18-65 years; (2) 23.0 ≦ BMI < 32.5 kg/m^2^; (3) waist circumference ≧ 80 cm; (4) Willingness to maintain their daily eating habits, daily routine, and exercise throughout the trial, and no intention to change their place of residence during the study; (5) understanding of the research procedures and signed the informed consent form. Exclusion criteria included gastrointestinal disorders (e.g., inflammatory bowel disease, irritable bowel syndrome, gastroesophageal reflux, Crohn's disease, or a history of bariatric surgery), recent acute gastroenteritis, constipation or diarrhea, mental disorders (e.g., depression or schizophrenia), cardiovascular diseases (e.g., hypertension), and allergies or sensitivities to the trial product. Participants with extreme eating habits, significant weight changes (> 4.5 kg in 3 months), recent antibiotic or antifungal use, or use of medications affecting gastrointestinal function (e.g., probiotics, laxatives, or NSAIDs) were excluded. Additional exclusions included exposure to unregistered medicines, alcohol or drug abuse, and female-specific conditions such as significant gastrointestinal symptoms during menstruation, irregular menstruation, pregnancy, planned pregnancy, or breastfeeding. Participants who became pregnant during the study were also excluded. A total of 70 participants were randomized in a 1:1 ratio to the placebo group or the AKK formula group (35 per group). Participants were instructed to take one sachet of powder daily for 8 weeks and attended follow-up visits at weeks 0, 4, and 8 for anthropometric measurements, blood tests, and stool analysis. Of these, 3 withdrew because of COVID-19 infection, leaving 67 participants who completed follow-up. Among these 67 participants, 17 did not meet the predefined compliance criteria and were therefore excluded from the per-protocol (PP) analysis. The trial was initially designed for 35 participants per group based on an a priori power analysis. The final PP population included 25 participants in each group.

### AKK supplement formulation

The AKK formula powder sachet was manufactured using a three-step process. Initially, fermented grape powder, fructooligosaccharide, and lactitol were combined in a specific ratio (w/w). This mixture was subsequently passed through a 30-mesh sieve. The sieved components were transferred to a high-capacity blender powder mixer and homogenized for 15 minutes. Throughout the manufacturing process, environmental conditions were maintained at approximately 20°C and 40% relative humidity. Quality control measures included analysis of moisture content, heavy metal presence, and microbiological testing. The final product was packaged in aluminum foil pouches and stored at room temperature. All procedures were conducted in compliance with good manufacturing practices to prevent cross-contamination. Table 1 shows that the AKK formula contained fermented grape powder, fructooligosaccharides, lactitol, indigestible maltodextrin, crystalline maltitol powder, calcium carbonate, citric acid, and steviol glycosides. The placebo contained indigestible maltodextrin, crystalline maltitol powder, calcium carbonate, citric acid, and steviol glycosides. The placebo and AKK formula were packaged in the same appearance, shape, and size. Subjects took one sachet of powder daily for 8 consecutive weeks. Each sachet was mixed with 150 mL of cold water before consumption.

### Blood analysis

Subjects were required to fast for at least 8 hours before blood sample collection. The collected blood was centrifuged in a refrigerated centrifuge (Hettich Zentrifugen, Universal 320R, Germany) at 3,000 rpm and 4°C for 20 minutes to separate the upper plasma from the lower blood cells. The plasma was aliquoted into microcentrifuge tubes for storage. The samples were then sent to Lezen Laboratory for analysis of blood glucose, insulin, the homeostasis model assessment of insulin resistance (HOMA-IR), calculated as fasting insulin (µU/mL) × fasting glucose (mg/dL)/22.5, high-density lipoprotein cholesterol (HDL-C), low-density lipoprotein cholesterol (LDL-C), glutamate-oxaloacetate transaminase (GOT), glutamate-pyruvate transaminase (GPT), blood urea nitrogen (BUN), creatinine, lactate dehydrogenase (LDH), chloride, calcium, phosphorus, and magnesium. Hematological tests included white blood cell (WBC), neutrophils, lymphocytes, monocytes, eosinophils, basophils, red blood cells (RBC), hemoglobin, hematocrit, mean cell volume (MCV), mean corpuscular hemoglobin (MCH), mean corpuscular hemoglobin concentration (MCHC), and platelet counts. All samples were stored at -80°C until analysis.

### Anthropometric assessments

Waist circumference was measured with the subject standing upright using a flexible tape to the nearest centimeter. For women, the measurement was taken at the narrowest point between the chest and hips, while for men, it was measured at the level of the umbilicus. Hip circumference was measured at the widest part of the buttocks, aligned with the level of the greater trochanters (hip bones), and recorded in centimeters. The Waist-to-Hip Ratio (WHR) was calculated by dividing the waist circumference by the hip circumference, offering insight into body fat distribution. WHR serves as an important indicator of health risks associated with body fat distribution, complementing other body measurements in health assessments and weight management strategies.

### Fecal sample collection

Participants were instructed to attach the sampling paper to the toilet seat in an appropriate position for fecal collection. After defecation, the cap of the collection tube, which was equipped with an attached fecal collection stick, was unscrewed, and a single scoop of the fecal sample was collected and placed into the tube. The cap was then tightly secured, and the tube was placed in a transparent plastic bag. The tube was gently shaken to ensure that the fecal sample was adequately immersed in the preservation solution. Collected samples were stored at room temperature and returned within one week of collection.

### *A. muciniphila* by species-specific qPCR

Stool samples were collected at home using a validated collection kit (BIOTOOLS Co., Ltd.), transferred into 1.5 mL tubes, and stored at -80 °C until analysis. Genomic DNA was extracted using the QIAamp PowerFecal kit (Qiagen) and quantified to ensure consistent input across samples. The relative abundance of *A. muciniphila* was determined using quantitative PCR (qPCR) with species-specific primers, while total bacterial load was measured using universal 16S rRNA primers. Reactions were prepared with KAPA SYBR FAST qPCR Master Mix (2×, KK4600) and run on a Bio-Rad CFX Connect system. Each 10 µL reaction contained 5 µL of master mix, 1 µL of each primer (2 µM), 2 µL of DNA template, and nuclease-free water. The thermal cycling conditions were 95 °C for 3 min, followed by 40 cycles of 95 °C for 3 s and 60 °C for 30 s, and a final melt curve analysis from 65 °C to 95 °C at 0.5 °C increments. All reactions were performed in triplicate, including non-template controls, and Cq values were verified using CFX Maestro software. Primer sequences were: *A. muciniphila* forward 5'-CAGCACGTGAAGTGGGGAC-3' and reverse 5'-CCTTGCGGTTGGCTTCAGAT-3'; universal 16S forward 5'-GTGYCAGCMGCCGCGGTAA-3' and reverse 5'-GGACTACNVGGGTWTCTAAT-3'. An inter-run calibrator (sample 24-3) was used to monitor plate-to-plate consistency. The relative abundance of *A. muciniphila* was calculated using the comparative quantification method, where ΔCq = Cq_{Akk} - Cq_{16S}, and relative abundance = 2^-ΔCq. To correct for potential plate effects, Cq values were normalized by subtracting the per-plate median before ΔCq calculation. Statistical analyses were performed using the Wilcoxon signed-rank test for within-group comparisons (Week 8 vs. Week 0) and rank-based or permutation tests for between-group differences.

### 16S rRNA gene amplicon profiling

For microbiome community profiling, the V3-V4 region of the bacterial 16S rRNA gene was amplified following the standard Illumina 16S Metagenomic Sequencing Library preparation protocol. In brief, approximately 12.5 ng of genomic DNA from each sample was used as the template for PCR amplification with KAPA HiFi HotStart ReadyMix (Roche) to ensure high fidelity. The primer pair used targeted the conserved V3-V4 region (forward 5'-CCTACGGGNGGCWGCAG-3'; reverse 5'-GACTACHVGGGTATCTAATCC-3'). The amplification conditions consisted of an initial denaturation at 95 °C for 3 min, followed by 25 cycles of 95 °C for 30 s, 55 °C for 30 s, and 72 °C for 30 s, with a final extension at 72 °C for 5 min. PCR products were examined on a 1.5% agarose gel, and fragments showing a clear band around 500 bp were purified using AMPure XP magnetic beads. A second PCR was then performed to attach Illumina adapter sequences and dual indices (Nextera XT), allowing sample multiplexing. The indexed libraries were checked for quality and concentration using Qubit 4.0 fluorometry and Qsep100 fragment analysis. After quantification, all libraries were pooled in equal amounts and subjected to paired-end sequencing (2 × 300 bp) on an Illumina MiSeq platform. All samples were processed simultaneously using a single sequencing run with uniform library preparation and equimolar pooling to avoid between-batch variability. Stool specimens were collected in a room-temperature stabilizer according to the kit instructions, stored at room temperature for ≤ 7 days, and transferred to -80 °C upon receipt to minimize pre-analytical variation. After quality control and chimera removal, the median effective sequencing depth was 49,523 reads per sample (minimum 21,348), ensuring sufficient coverage for downstream α- and β-diversity analyses.

### Microbiome bioinformatics

Raw paired-end sequencing reads were analyzed using QIIME 2 (version 2025.4). After merging paired reads, low-quality sequences and chimeras were removed to ensure data integrity. High-quality reads were clustered into operational taxonomic units (OTUs) at 97% sequence identity using the VSEARCH algorithm. Representative sequences were aligned with MAFFT, and a phylogenetic tree was constructed using FastTree and midpoint-rooted for downstream analysis. Taxonomic classification was performed with a Naïve Bayes classifier trained on the SILVA 138.1 database restricted to the V3-V4 region of the 16S rRNA gene. To ensure comparability, sequencing depth was normalized by rarefying the OTU table to 20,000 reads per sample using qiime feature-table rarefy. All 134 samples exceeded this threshold (minimum effective depth 21,348), and rarefaction curves (observed OTUs versus sequencing depth) plateaued at approximately 15,000-20,000 reads, supporting this choice. Differential abundance analyses were performed using *metagenomeSeq* with cumulative sum scaling (CSS) normalization without rarefaction to minimize compositional bias 11. Alpha diversity, including the Shannon index, observed OTU richness, and Faith's phylogenetic diversity, was calculated for each sample, and group comparisons at baseline and Week 8 were assessed using Welch's t-tests with Hedges' g as the effect size. Beta diversity was evaluated based on Bray-Curtis dissimilarities and visualized by principal coordinates analysis (PCoA), with group differences determined by pairwise PERMANOVA (999 permutations) followed by false discovery rate (FDR) correction. Differentially abundant taxa were identified using metagenomeSeq with cumulative sum scaling (CSS) normalization and FDR adjustment 11. Functional profiles were predicted using PICRUSt2 to infer MetaCyc pathways, with the Nearest Sequenced Taxon Index (NSTI) summarizing prediction quality, and pathway-level comparisons adjusted for multiple testing. Exploratory analyses were further supported by LEfSe, and key findings were confirmed by FDR-corrected statistical tests 12. This integrated workflow provided a comprehensive assessment of microbial composition, diversity, and functional potential across groups and time points.

### Statistical analysis

Data processing and statistical analyses were performed using IBM SPSS Statistics version 22.0 and SAS Studio version 3.8. Group results are presented as mean ± standard deviation (mean ± SD). Within-group changes from baseline to subsequent time points were analyzed using paired t-tests, whereas between-group differences in the change from baseline (Δ₄₋₀ and Δ₈₋₀) were assessed using independent samples t-tests. To minimize the risk of false positives arising from multiple comparisons, p-values for between-group analyses were adjusted using the false discovery rate (FDR) procedure. A mixed-design two-way analysis of variance (ANOVA) was used to assess the group, time, and group × time interaction effects. When a significant main effect of group was identified, post hoc independent samples t-tests were conducted. For significant main effects of time, one-way repeated-measures ANOVA followed by least significant difference (LSD) post hoc tests was applied to evaluate pairwise differences in body composition indices. Paired samples t-tests were additionally used to assess within-group changes in metabolic and liver/kidney function parameters. All analyses were conducted according to the prespecified statistical analysis plan without baseline covariate adjustment, as baseline characteristics were balanced between groups. Statistical significance was defined as *p* < 0.05. The main analyses presented in this study were conducted in the per-protocol population (n = 25 per group), given the importance of adequate adherence for exposure to the intervention. In addition, a supplementary post-randomization available-case analysis including the 67 participants who completed follow-up was conducted as a robustness assessment.

## Results

### Assessment of safety profile of AKK formula

**Figure 1:**
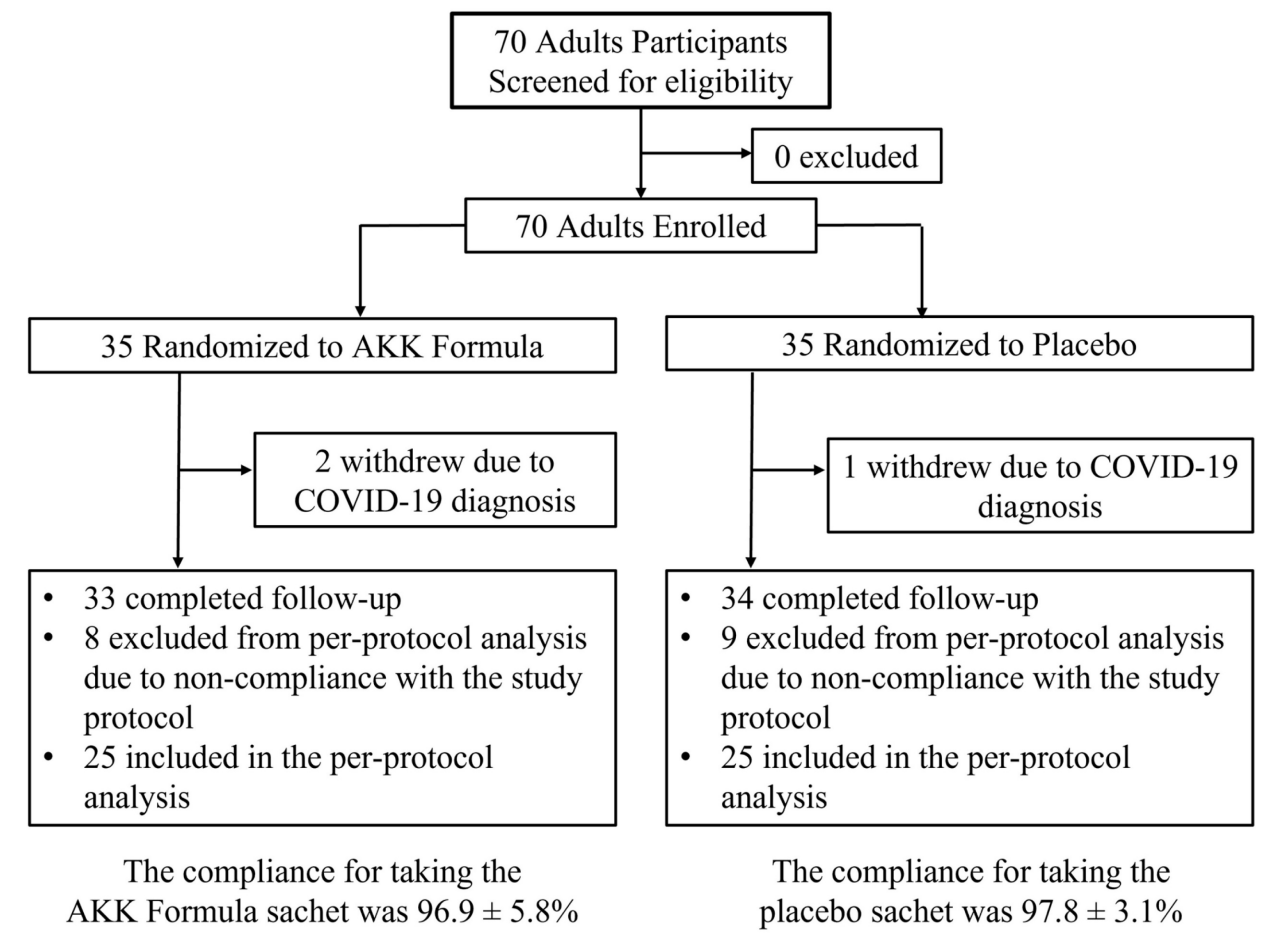
Clinical trial flow chart. Of the 70 randomized participants, 3 withdrew because of COVID-19 infection. Among the 67 participants who completed follow-up, 17 were excluded from the per-protocol analysis because they did not meet the predefined compliance criteria, resulting in 50 participants (25 per group) included in the per-protocol analysis.

Of the 70 randomized participants, 3 withdrew due to COVID-19 infection, leaving 67 participants who completed follow-up. Among these, 17 participants did not meet the predefined compliance criteria and were excluded from the per-protocol (PP) analysis (Fig. 1). Therefore, the primary analyses presented in the main text were conducted in the PP population (n = 25 per group), which included participants with adequate adherence to the supplementation protocol. Supplementary post-randomization available-case analyses, including 67 participants who completed follow-up, are provided in Supplementary Tables S6-S9 as robustness assessments.

Table 2 shows no significant differences in baseline characteristics between the two groups in the PP population. Table 3 summarizes serum markers of liver and kidney function, as well as inflammatory indicators, following 8 weeks of AKK formula consumption. Based on these safety assessments, no abnormal liver or kidney function or inflammatory reactions were observed, supporting the safety profile of the formula for consumption.

### AKK formula increased the relative abundance of *A. muciniphila*

The AKK formula was associated with a significant increase in the relative abundance of *A. muciniphila*. As summarized in Table 4, quantitative PCR analysis demonstrated a significant rise in the relative proportion of *A. muciniphila* from Week 0 to Week 8 in the AKK group (Wilcoxon *p* = 0.003), corresponding to a median increase of approximately 2.1-fold relative to baseline. In addition, a significantly greater proportion of participants in the AKK group exhibited at least a 10% increase in the relative abundance of *A. muciniphila* compared with the placebo group (82.6% vs. 43.5%, Fisher's exact *p* < 0.05). These findings indicate that AKK supplementation was associated with a consistent shift toward a higher relative representation of *A. muciniphila* within the gut microbial community, rather than reflecting isolated changes in a small subset of individuals.

### Effects of the AKK formula on anthropometric parameters

As shown in Table 5, significant between-group differences were observed for body weight and BMI at week 8. Compared with the placebo group, the AKK formula group demonstrated greater reductions in body weight (between-group Δ₈-₀ mean difference: -1.42 kg; 95% CI: -1.98 to -0.87; FDR-adjusted *p* = 0.0009) and BMI (between-group Δ₈-₀ mean difference: -0.50 kg/m²; 95% CI: -0.69 to -0.32; FDR-adjusted *p* = 0.0009). In contrast, although within-group reductions in waist and hip circumference were observed in the AKK formula group, the between-group differences for waist circumference (FDR-adjusted *p* = 0.4812) and hip circumference (FDR-adjusted *p* = 0.2527) were not statistically significant. No significant between-group differences were observed for waist-to-hip ratio (WHR).

### Effects of the AKK formula on blood lipid parameters

As shown in Table 6, no statistically significant between-group differences were observed in blood lipid or metabolic parameters after the 8-week intervention. Total cholesterol levels remained relatively stable in the AKK formula group, while a modest increase was observed in the placebo group; however, the between-group difference was not statistically significant (FDR-adjusted *p* = 0.2527). LDL-C showed a small decrease in the AKK formula group and an increase in the placebo group. Although the between-group difference reached nominal significance (*p* = 0.0134), it did not remain significant after false discovery rate (FDR) adjustment (FDR-adjusted *p* = 0.0759). HDL-C increased slightly in both groups, with no significant between-group difference. Similarly, changes in glucose, insulin, and insulin resistance indices did not differ significantly between groups. Overall, these findings indicate that AKK formula supplementation was associated with relative stability of blood lipid and metabolic parameters during the study period, without evidence of statistically significant between-group effects.

### Effects of the AKK formula on gut microbial diversity and community-level features

**Figure 2:**
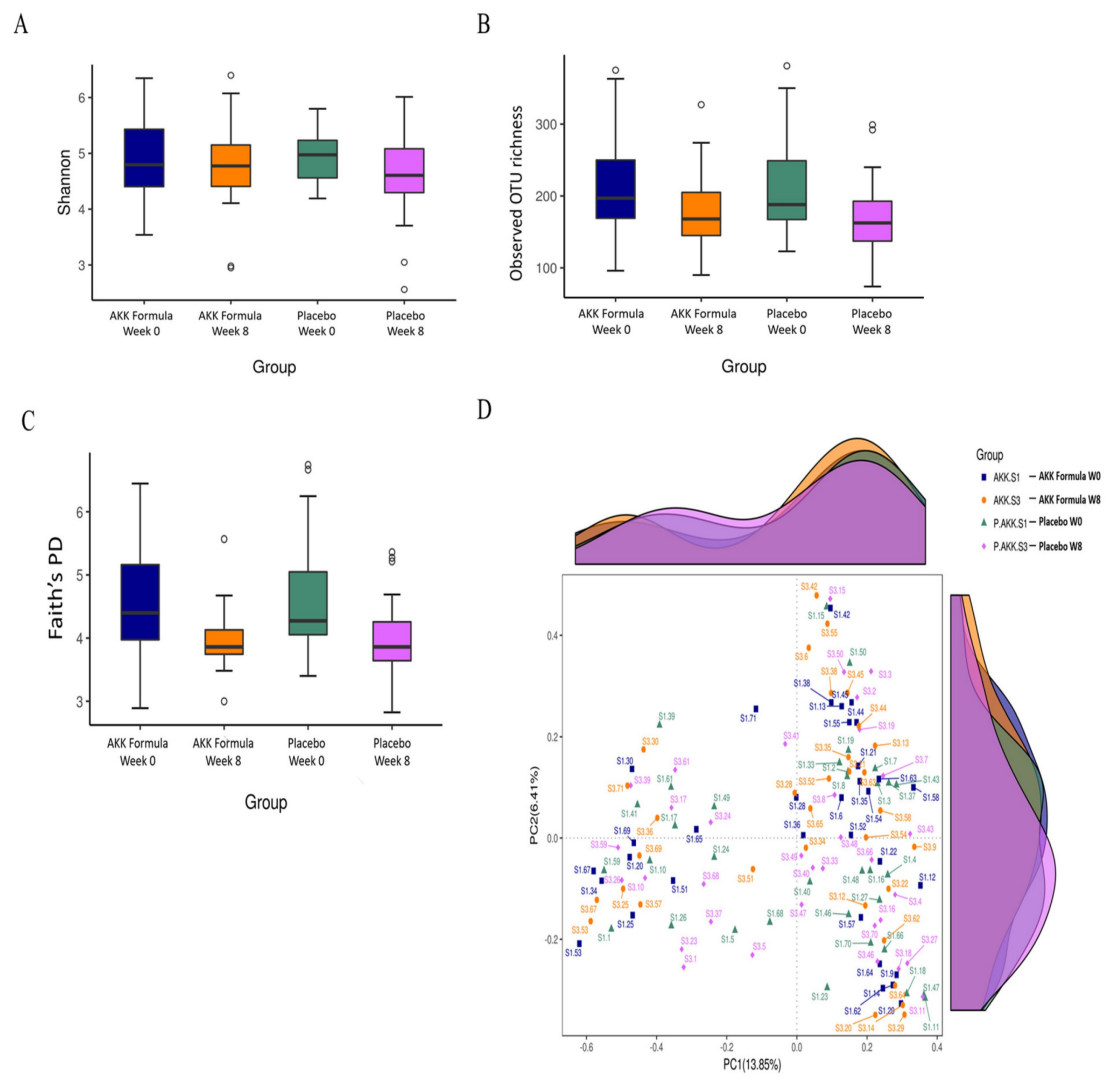
Gut microbiota diversity and community structure following AKK formula supplementation. Boxplots show (A) Shannon diversity index, (B) observed OTU richness, and (C) Faith's phylogenetic diversity (PD) in the AKK formula and placebo groups at baseline (Week 0) and after 8 weeks of intervention. (D) Principal coordinates analysis (PCoA) based on Bray-Curtis dissimilarities illustrates the similarity of gut microbial community structures across groups and time points.

To further characterize the impact of the AKK formula on gut microbiota composition and community-level features, microbial diversity and overall community structure were analyzed. At Week 8, measures of alpha diversity remained comparable between the AKK formula and placebo groups. The Shannon diversity index (mean ± SD: 4.77 ± 0.77 vs. 4.56 ± 0.77; *p* = 0.33; Hedges' g = 0.27, small) showed no statistically significant difference, and similar results were observed for the number of observed species (180.3 ± 52.5 vs. 172.2 ± 54.1; *p* = 0.27; g = 0.15) and Faith's phylogenetic diversity (3.98 ± 0.49 vs. 4.00 ± 0.63; *p* = 0.92; g = -0.03). Baseline alpha diversity metrics were also comparable between groups (e.g., Shannon index: 4.82 ± 0.67 vs. 4.99 ± 0.45; *p* = 0.30; g = -0.29), indicating similar microbial diversity at study entry (Fig. 2A-2C). Analysis of beta diversity using principal coordinates analysis (PCoA) based on Bray-Curtis distances (Fig. 2D) demonstrated substantial overlap in overall microbial community composition between groups and across time points, suggesting a largely stable community structure over the 8-week intervention period. Consistently, pairwise PERMANOVA analyses revealed minimal between-group variation (all R² < 0.03), with no statistically significant differences after FDR correction (Supplementary Table S4). Predictive functional profiling using PICRUSt2 showed acceptable prediction quality for human fecal samples, as indicated by low Nearest Sequenced Taxon Index (NSTI) values (median 0.064, interquartile range 0.048-0.077). However, no MetaCyc pathways remained significant after multiple-testing correction (Supplementary Table S5). Collectively, these findings suggest that the AKK formula was associated with taxon-specific shifts, including an increased relative abundance of *A. muciniphila*, while overall microbial diversity, community structure, and predicted metabolic functions at the community level remained stable during the intervention period.

### AKK formula was associated with selective changes in gut microbiota composition

**Figure 3:**
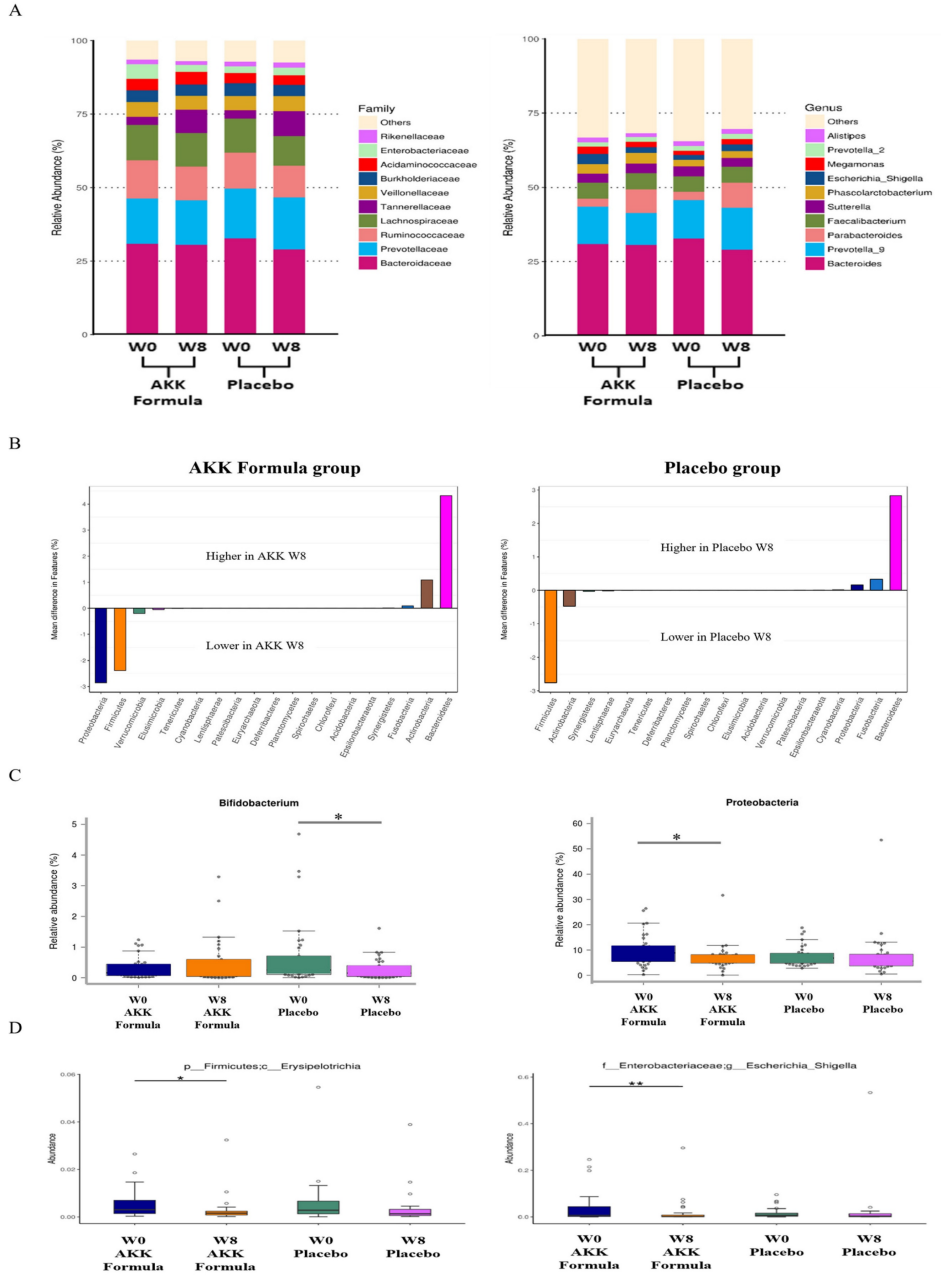
AKK formula was associated with selective changes in gut microbiota composition. Subjects received placebo or the AKK formula for 8 weeks, and stool samples were analyzed by 16S rRNA gene sequencing. (A) Family-level relative abundance. (B) Genus-level relative abundance. (C) Relative abundance of *Bifidobacterium* and *Proteobacteria* at weeks 0 and 8. (D) Relative abundance of *Erysipelotrichia* and *Escherichia-Shigella* at weeks 0 and 8. * and ** denote *p* < 0.05 and *p* < 0.01 vs. baseline.

NGS results indicated that the AKK formula did not markedly alter the overall structure of the indigenous gut microbiota, suggesting selective modulation of specific taxa without broad disruption of microbial diversity or predicted community function (Fig. 3A,B). *Bifidobacterium*, a genus within the phylum *Actinobacteria*, has been associated with weight management, reduced adiposity, and improved lipid metabolism in previous studies 13,14, whereas *Proteobacteria* has been consistently reported as an obesity-associated phylum. Proteobacteria, which includes many human-associated pathogens, may serve as a microbial marker of disease 15. After 8 weeks of AKK formula supplementation, *Bifidobacterium* increased, whereas Proteobacteria decreased significantly compared with baseline (week 0) in the treatment group, indicating taxon-specific changes in gut microbial composition (Fig. 3C). Elevated levels of Erysipelotrichia have been associated with gut inflammation and metabolic disturbances 16.

*Escherichia-Shigella* has been identified in previous studies as a taxon associated with obesity and non-alcoholic fatty liver disease (NAFLD) 17. After 8 weeks of AKK formula supplementation, the relative abundance of *Erysipelotrichia* and *Escherichia-Shigella* decreased significantly compared with baseline (week 0) in the treatment group (Fig. 3D). Within the AKK formula group, *Bifidobacterium* increased and *Proteobacteria*, *Erysipelotrichia*, and *Escherichia-Shigella* decreased relative to baseline, indicating selective shifts in gut microbial composition.

### AKK formula and gastrointestinal symptoms

The Gastrointestinal Symptom Rating Scale (GSRS) includes 15 items grouped into five symptom clusters: reflux, abdominal pain, indigestion, diarrhea, and constipation 18. As shown in Table 7, total GSRS scores tended to decrease in the AKK formula group, whereas no clear change was observed in the placebo group.

## Discussion

The AKK formula demonstrated a favorable safety profile, with no adverse effects observed on liver or kidney function. Quantitative PCR analysis showed a significant increase in the relative abundance of *A. muciniphila* relative to total bacteria, along with a higher proportion of responders in the AKK formula group, consistent with previous evidence indicating that dietary interventions can enhance the abundance of *A. muciniphila* in the gut microbiota 19-23. In parallel, changes in anthropometric measures and blood lipid parameters were modest and did not consistently demonstrate statistically significant between-group differences. In addition to the increase in *A. muciniphila*, the AKK formula was associated with reductions in the relative abundance of several taxa, including *Proteobacteria*, *Erysipelotrichia*, and *Escherichia-Shigella*, taxa that have previously been associated with inflammation, obesity, or metabolic dysfunction 15-17. Despite these targeted microbial shifts, α-diversity remained stable and β-diversity exhibited only minor, non-significant variation, suggesting that the intervention selectively modulated specific bacterial taxa without broadly altering overall microbial community structure.

Previous studies have consistently shown that dietary interventions can modulate the abundance of Akkermansia muciniphila. Calorie restriction in adults has been associated with a significant increase in *A. muciniphila* levels 24. Certain polyphenol-rich foods and extracts, such as pomegranate extract and grape powder, have been reported to promote the growth of *A. muciniphila* in the gut, with corresponding associations with body composition and gut-related parameters in animal models 19. In preclinical studies, supplementation with prebiotics such as oligofructose markedly increased *A. muciniphila* abundance in genetically obese mice, in some cases by more than 100-fold 20. Restoration of *A. muciniphila* levels through prebiotic feeding has been associated with favorable changes in body composition and metabolic markers in mouse models 21. Similarly, high-fiber diets rich in non-digestible prebiotic fibers have been shown to enhance *A. muciniphila* abundance in the gut microbiome 22. Specific dietary phytochemicals, including curcumin and epigallocatechin gallate (EGCG), have also been demonstrated to increase *A. muciniphila* levels in murine studies 23. Mechanistic investigations suggest that *A. muciniphila* plays a role in host-microbe interactions at the intestinal mucus layer. The presence of viable *A. muciniphila* has been implicated in the regulation of mucus turnover and maintenance of intestinal barrier function 25. In animal models, administration of live *A. muciniphila* has been associated with attenuation of high-fat diet-induced metabolic disturbances, including changes in body composition, metabolic endotoxemia, inflammation, and insulin sensitivity, whereas heat-killed cells did not produce comparable effects 22,26. Several preclinical studies have reported that specific *A. muciniphila*-derived components, including the membrane protein Amuc_1100 and the secreted protein P9, may modulate host metabolic parameters and gut microbial balance in animal models 27. These findings provide important mechanistic insights from experimental systems, supporting the concept that *A. muciniphila* can influence host physiology under controlled conditions. However, such mechanisms were not directly assessed in the present study, and their relevance to human clinical outcomes remains to be fully elucidated 28. Previous experimental work has suggested that *A. muciniphila*-derived factors may influence host metabolic pathways through multiple mechanisms. In murine models, the secreted protein P9 has been shown to interact with intercellular adhesion molecule 2 (ICAM-2) and promote macrophage polarization toward an M2 phenotype via an interleukin-6-dependent pathway, which has been associated with changes in glucose homeostasis and insulin sensitivity 24. In addition, *A. muciniphila* metabolic activity has been reported to interact with host lipid-related metabolites and inflammatory signaling pathways in animal studies 19. Other mechanistic investigations have proposed roles for *A. muciniphila* in modulating endotoxin levels, influencing short-chain fatty acid (SCFA) profiles, and affecting fatty acid oxidation in intestinal and adipose tissues 29. Importantly, SCFAs, microbial proteins, and related metabolic mediators were not measured in this clinical trial, and therefore these mechanisms should be interpreted as contextual background rather than evidence derived from the current data. The present study does not establish a direct causal link between increased *A. muciniphila* relative abundance and metabolic improvement in humans. Beyond host metabolic pathways, enrichment of *A. muciniphila* has also been associated with broader changes in gut microbiota composition in previous studies, including shifts in the relative abundance of major bacterial phyla such as *Firmicutes*, *Bacteroidetes*, *Actinobacteria*, and *Euryarchaeota* 30. *A. muciniphila* has been reported to coexist with multiple commensal taxa, suggesting potential ecological interactions within the gut microbial ecosystem 31. Consistent with this literature, changes in gut microbiota composition have been linked to metabolic phenotypes in observational studies 32-34. Collectively, these findings underscore the complex and multifactorial relationships between gut microbiota composition and host metabolic status, while highlighting the need for cautious interpretation when extrapolating mechanistic or associative evidence from preclinical and observational studies to human clinical outcomes 35.

This study has several limitations that should be acknowledged. First, the relatively small sample size and the 8-week intervention period limit the generalizability of the findings and preclude conclusions regarding long-term effects of the AKK formula. Although statistically significant between-group differences were observed for body weight and BMI, the magnitude of these changes was modest (approximately <1 kg reduction in body weight). Changes in waist and hip circumference were observed primarily in within-group analyses and did not demonstrate statistically significant between-group differences, suggesting limited clinical relevance. Second, although participants were instructed to maintain their usual diet and lifestyle throughout the study, adherence could not be objectively verified. While daily intake of calories, carbohydrates, protein, fat, and fiber was recorded and is summarized in Supplementary Table S3, detailed dietary patterns and physical activity levels were not systematically assessed. Consequently, unmeasured dietary or lifestyle factors may have influenced gut microbiota composition and metabolic outcomes. Third, the abundance of Akkermansia muciniphila was quantified using species-specific quantitative PCR (qPCR) and expressed as relative abundance (2^-ΔCq) normalized to total bacterial 16S rRNA gene copies. Because the exported dataset did not include standard curve data or extraction efficiency metadata, absolute bacterial counts (e.g., copies per gram of stool) could not be determined. Although normalization procedures were applied to minimize inter-plate variability, relative abundance measurements may still be influenced by variations in total bacterial load. Finally, 16S rRNA gene sequencing targeting the V3-V4 region provides limited taxonomic resolution at the species or strain level. Future studies with larger sample sizes and longer follow-up periods should incorporate absolute quantification approaches, such as digital PCR, as well as higher-resolution sequencing techniques, including shotgun metagenomics, to validate species- and strain-level changes and to further elucidate the mechanistic effects of the AKK formula.

## Conclusion

Our results indicate that the AKK formula was well tolerated over the 8-week intervention period and was associated with an increased relative abundance of *A. muciniphila* and targeted modulation of gut microbiota composition. Changes in anthropometric and metabolic parameters were modest, while the microbiota-related findings were more pronounced. Overall, the findings support the short-term safety of the AKK formula and provide exploratory evidence of its ability to selectively influence gut microbiota profiles. Further studies with larger sample sizes, longer follow-up durations, and mechanistic investigations are warranted to clarify the biological significance and potential clinical implications of these microbiota changes.

## Supplementary Material

Supplementary tables.

## Figures and Tables

**Table 1:** Ingredients of the AKK formula and placebo

AKK formula	Placebo
Fermented grape powder (grape extract, glucose, isomalto oligosaccharide, vinegar, maltodextrin)	-
Fructooligosaccharides	-
Lactitol	-
Indigestible maltodextrin	Indigestible maltodextrin
Crystalline Maltitol Powder	Crystalline Maltitol Powder
Calcium Carbonate	Calcium Carbonate
Citric Acid	Citric Acid
Steviol Glycosides	Steviol Glycosides
Flavor	Flavor

**Table 2:** Baseline anthropometric and biochemical characteristics

Characteristics	AKK formula (n = 25)	Placebo (n = 25)	Reference range
Female / Male (%)	44 / 56	40 / 60	—
Age (years)	39.1 ± 13.7	33.0 ± 11.9	—
Weight (kg)	80.6 ± 15.2	78.6 ± 11.4	—
BMI (kg/m²)	28.4 ± 3.2	28.3 ± 3.5	—
Waist circumference (cm)	96.79 ± 11.7	95.3 ± 9.0	—
Hip circumference (cm)	103.3 ± 6.69	103.6 ± 5.5	—
Glucose (mg/dL)	90.4 ± 14.1	85.7 ± 4.9	74-109
GOT (U/L)	18.8 ± 7.4	19.3 ± 6.1	8-39
GPT (U/L)	24.8 ± 18.4	28.2 ± 20.1	<41
BUN (mg/dL)	13.1 ± 3.0	12.4 ± 2.7	7-25
Creatinine (mg/dL)	0.70 ± 0.17	0.74 ± 0.17	0.6-1.3
Triglyceride (mg/dL)	111.3 ± 64.5	133.2 ± 97.2	< 150
Total cholesterol (mg/dL)	197.6 ± 42.1	187.3 ± 40.0	< 200
HDL-C (mg/dL)	54.0 ± 13.7	51.2 ± 9.5	≥ 40 (M), ≥ 50 (F)
LDL-C (mg/dL)	126.2 ± 38.2	115.4 ± 33.7	< 130

Data are presented as mean ± SD or n (%). Abbreviations: BMI, body mass index; GOT, glutamate-oxaloacetate transaminase; GPT, glutamate-pyruvate transaminase; BUN, blood urea nitrogen; HDL-C, high-density lipoprotein cholesterol; LDL-C, low-density lipoprotein cholesterol.

**Table 3:** Safety assessment of the AKK formula after 8 weeks of supplementation

Parameter	AKK formula (n = 25)	Placebo (n = 25)	Reference range
Liver function			
GOT (U/L)	18.8 ± 7.4 → 20.3 ± 7.1	19.3 ± 6.1 → 22.5 ± 12.2	8 - 39
GPT (U/L)	24.8 ± 18.4 → 28.2 ± 20.1	28.2 ± 20.1 → 31.2 ± 32.2	< 41
Total bilirubin (mg/dL)	0.6 ± 0.2 → 0.7 ± 0.3	0.7 ± 0.3 → 0.6 ± 0.2	0.3 - 1.0
Kidney function			
BUN (mg/dL)	13.1 ± 3.0 → 12.4 ± 2.7	12.4 ± 2.7 → 13.9 ± 2.7	7 - 25
Creatinine (mg/dL)	0.70 ± 0.17 → 0.74 ± 0.17	0.74 ± 0.17 → 0.79 ± 0.15	0.6 - 1.3
Uric acid (mg/dL)	5.8 ± 1.4 → 5.5 ± 1.7	5.5 ± 1.7 → 5.7 ± 1.4	M: 4.4 - 7.6 / F: 2.3 - 6.6
Thyroid and metabolism			
TSH (μIU/mL)	2.1 ± 1.2 → 1.7 ± 1.3	2.1 ± 1.1 → 2.3 ± 0.9	0.38 - 5.33
Hematology			
RBC (×10⁶/µL)	4.8 ± 0.4 → 4.8 ± 0.4	4.9 ± 0.4 → 5.0 ± 0.5	M: 4.5-6.0 / F: 4.0-5.2
Hemoglobin (g/dL)	13.8 ± 2.1 → 13.8 ± 2.1	14.2 ± 1.3 → 14.4 ± 1.4	M: 13.5-17.5 / F: 11.5-15.5
Platelet count (×10³/µL)	262 ± 68 → 258 ± 78	278 ± 67 → 285 ± 71	130 - 400

Data are presented as mean ± SD. No clinically relevant adverse changes were observed after 8 weeks of supplementation. Abbreviations: GOT, glutamate-oxaloacetate transaminase; GPT, glutamate-pyruvate transaminase; BUN, blood urea nitrogen; TSH, thyroid-stimulating hormone; RBC, red blood cell.

**Table 4:** Relative abundance of *A. muciniphila* in fecal samples determined by qPCR

Characteristics	AKK formula (n = 25)		Placebo (n = 25)	
	Week 0	Week 8	Week 0	Week 8
Relative abundance of *A. muciniphila*	1.0 fold	2.12 fold	1.0 fold	0.54 fold
The proportion of subjects who experienced an increase in *A. muciniphila* of more than 10%	-	82.6%^#^	-	43.50%

Data are presented as fold change relative to baseline. # *p* < 0.05 compared with the placebo group. Statistical analysis for responder proportion was performed using Fisher's exact test, with a ≥10% increase in *A. muciniphila* set as the threshold.

**Table 5:** Effects of the AKK formula on anthropometric measurements

Characteristics	AKK formula (n = 25)					Placebo (n = 25)						
	Week 0	Week 4	Week 8	Δ₄₋₀ (mean, 95% CI)	Δ₈₋₀ (mean, 95% CI)	Week 0	Week 4	Week 8	Between-group Δ₄₋₀ (mean diff, 95% CI)	Between-group Δ₈₋₀ (mean diff, 95% CI)	p-value (Δ₈₋₀)	p-value (Δ₈₋₀) (FDR-adjusted)
Weight (kg)	80.55±15.20	80.14±15.08^*^	79.72±14.95^*#^	-0.41 (-0.75, -0.07)	-0.82 (-1.25, -0.4)	78.55±11.41	78.64±11.36	79.15±11.38^*^	-0.50 (-0.97, -0.03)	-1.42 (-1.98, -0.87)	<.0001	0.0009
BMI (kg/m^2^)	28.41±3.18	28.27±3.14^*^	28.13±3.14^*#^	-0.14 (-0.26, -0.03)	-0.29 (-0.43, -0.14)	28.34±3.45	28.37±3.43	28.55±3.39^*^	-0.17 (-0.34, -0.01)	-0.50 (-0.69, -0.32)	<.0001	0.0009
Waist (cm)	96.79±11.72	96.17±11.71	95.89±11.62^*^	0.34 (-0.93, 1.61)	-0.39 (-1.66, 0.89)	95.33±9.01	94.92±8.60	95.72±8.72	-0.32 (-2.10, 1.46)	-0.91 (-2.92, 1.10)	0.3680	0.4812
Hip (cm)	103.29±6.69	103.22±6.65	102.86±6.52^*^	-1.64 (-2.75, -0.53)	-1.78 (-2.60, -0.96)	103.56±5.53	103.44±5.68	103.69±5.64	-0.42 (-2.73, 1.89)	-1.08 (-2.42, 0.26)	0.1104	0.2527
WHR (%)	0.933±0.066	0.930±0.06	0.930±0.066	0.02 (0.002, 0.03)	0.01 (-0.003, 0.03)	0.913±0.047	0.915±0.040	0.921±0.039	0(-0.02, 0.02)	0(-0.02, 0.02)	0.9656	0.9656

Data are presented as mean ± SD. Δ₄₋₀ and Δ₈₋₀ indicate the changes from week 0 to week 4 and from week 0 to week 8, respectively. * *p* < 0.05 compared with week 0 (baseline); # *p* < 0.05 compared with the placebo group. P-values for between-group comparisons were adjusted using the false discovery rate (FDR) procedure. Abbreviations: BMI, body mass index; WHR, waist-to-hip ratio; CI, confidence interval.

**Table 6:** Effects of the AKK formula on blood lipids and metabolic variables

Characteristics	AKK formula (n = 25)			Placebo (n = 25)				
	Week 0	Week 8	Δ₈₋₀ (mean, 95% CI)	Week 0	Week 8	Between-group Δ₈₋₀ (mean diff, 95% CI)	p-value (Δ₈₋₀)	p-value (Δ₈₋₀) (FDR-adjusted)
Total cholesterol (mg/dL)	197.56±42.1	197.08±46.5	-0.48 (-8.91, 7.95)	187.28±40.0	195.96±38.3^*^	-9.16 (-20.44, 2.12)	0.1091	0.2527
LDL-C (mg/dL)	126.20±38.17	125.88±40.02	-0.32 (-6.77, 6.13)	115.36±33.71	126.48±36.26^*^	-11.44 (-20.40, -2.48)	0.0134	0.0759
HDL-C (mg/dL)	54.0±13.7	55.1 ± 15.3	1.05 (-0.91, 3.01)	51.2±9.5	54.9±10.2^*^	-2.60 (-5.45, 0.26)	0.0735	0.2527
Glucose (mg/dL)	90.40±14.09	91.52±12.76	1.12 (-1.23, 3.47)	85.68±4.85	84.68±8.61^*^	2.12 (-2.16, 6.40)	0.3241	0.4591
Insulin (µU/mL)	15.88±9.32	13.46±6.23	-2.42 (-5.07, 0.22)	14.59±9.26	15.39±8.35	-3.23 (-7.24, 0.79)	0.1125	0.2527
Insulin resistance	3.63±2.3	3.12±1.8	-0.52 (-1.08, 0.05)	3.09±1.9	3.22±1.8	-0.64 (-1.51, 0.23)	0.1440	0.2542

Data are presented as mean ± SD. Δ₈₋₀ indicates the change from week 0 to week 8. * *p* < 0.05 compared with week 0 (baseline); # *p* < 0.05 compared with the placebo group. *P*-values for between-group comparisons were adjusted using the false discovery rate (FDR) procedure. Abbreviations: HDL-C, high-density lipoprotein cholesterol; LDL-C, low-density lipoprotein cholesterol; CI, confidence interval.

**Table 7:** Effects of the AKK formula on gastrointestinal symptom rating scale scores

Characteristics	AKK formula (n = 25)					Placebo (n = 25)						
	Week 0	Week 4	Week 8	Δ₄₋₀ (mean, 95% CI)	Δ₈₋₀ (mean, 95% CI)	Week 0	Week 4	Week 8	Between-group Δ₄₋₀ (mean diff, 95% CI)	Between-group Δ₈₋₀ (mean diff, 95% CI)	p-value (Δ₈₋₀)	p-value (Δ₈₋₀) (FDR-adjusted)
Total GSRS score	24.5 ± 6.9	23.6 ± 7.6	22.9 ± 5.6	-0.96 (-3.34, 1.42)	-1.60 (-3.29, 0.09)	26.1 ± 7.6	25.9 ± 6.5	26.4 ± 8.5	-0.76 (-4.35, 2.83)	-1.88 (-5.17, 1.41)	0.2561	0.3958
Abdominal pain	3.9 ± 1.1	3.9 ± 1.5	3.6 ± 1.0	0(-0.51, 0.51)	-0.24 (-0.71, 0.23)	3.9 ± 1.3	4.0 ± 1.4	4.2 ± 1.7	-0.16 (-0.95, 0.63)	-0.52 (-1.23, 0.19)	0.1495	0.2542
Reflux	2.6 ± 1.7	2.2 ± 0.7	2.4 ± 0.7	-0.32 (-0.99, 0.35)	-0.20 (-0.72, 0.32)	2.5 ± 1.0	2.3 ± 0.7	2.6 ± 0.9	-0.16 (-0.92, 0.60)	-0.28 (-0.98, 0.42)	0.4224	0.5129
Indigestion	8.3 ± 3.4	8.5 ± 4.6	8.3 ± 3.4	0.16 (-1.04, 1.36)	0(-0.83, 0.83)	9.5 ± 3.3	9.6 ± 3.4	9.4 ± 3.7	0.04 (-1.53, 1.61)	0.08 (-1.40, 1.56)	0.9137	0.9656
Diarrhea	5.1 ± 2.2	5.0 ± 2.2	4.6 ± 1.7	-0.16 (-0.85, 0.53)	-0.52 (-1.06, 0.02)	5.5 ± 2.9	5.2 ± 2.5	5.3 ± 2.4	0.12 (-1.24, 1.48)	-0.28 (-1.30, 0.74)	0.5816	0.6591
Constipation	4.6 ± 1.7	4.0 ± 1.4	4.0 ± 0.9	-0.64 (-1.30, 0.02)	-0.64 (-1.32, 0.04)	4.7 ± 2.1	4.7 ± 1.8	5.0 ± 2.5	-0.60 (-1.70, 0.50)	-0.88 (-1.99, 0.23)	0.1189	0.2527

Data are presented as mean ± SD. Δ₄₋₀ and Δ₈₋₀ indicate the changes from week 0 to week 4 and from week 0 to week 8, respectively. P-values for between-group comparisons were adjusted using the false discovery rate (FDR) procedure. Abbreviations: GSRS, Gastrointestinal Symptom Rating Scale; CI, confidence interval.

## Data Availability

The data supporting the findings of this study are available from the corresponding author upon reasonable request.

## Abbreviations

AKK formula: prebiotic formula promoting *A. muciniphila*
BMI: Body Mass Index
DNA: Deoxyribonucleic Acid
EGCG: Epigallocatechin Gallate
GPT: Glutamate-Pyruvate Transaminase
GOT: Glutamate-Oxaloacetate Transaminase
GSRS: Gastrointestinal Symptom Rating Scale
HDL-C: High-Density Lipoprotein Cholesterol
HOMA-IR: Homeostasis Model Assessment of Insulin Resistance
ICAM-2: Intercellular Adhesion Molecule 2
LDH: Lactate Dehydrogenase
LDL-C: Low-Density Lipoprotein Cholesterol
LDA: Linear Discriminant Analysis
MCV: Mean Cell Volume
MCH: Mean Corpuscular Hemoglobin
MCHC: Mean Corpuscular Hemoglobin Concentration
NGS: Next-Generation Sequencing
NSAIDs: Nonsteroidal Anti-Inflammatory Drugs
PCR: Polymerase Chain Reaction
qPCR: Quantitative Polymerase Chain Reaction
RBC: Red Blood Cell
SCFA: Short-Chain Fatty Acid
SPSS: Statistical Package for the Social Sciences
WHR: Waist-to-Hip Ratio
WBC: White Blood Cell
ZIG: Zero-Inflated Gaussian
