# Supplementary material for: The Effects of a Prebiotic Formula Promoting Akkermansia muciniphila (AKK) on Gut Health: A Single-Centre, Randomised Controlled Trial
Source: Int J Med Sci. 2026 Apr 23;23(6):1952–65. doi: 10.7150/ijms.125881 (PMC13181376; doi:10.7150/ijms.125881)
Supplement: Supplementary file 1 — Supplementary tables. [file ijmsv23p1952s1.pdf]

Supplementary Table S1. Baseline anthropometric and biochemical characteristics

Characteristics	AKK formula (n = 33)	Placebo (n = 34)	Reference range
Female / Male (%)	17 (52) / 16 (48)	16 (47) / 18 (53)	—
Age (years)	37.5 ± 13.6	35.3 ± 13.4	—
Weight (kg)	80.3 ± 14.8	77.6 ± 10.9	—
BMI (kg/m²)	28.4 ± 3.0	28.2 ± 3.2	—
Waist circumference (cm)	93.7 ± 9.1	92.0 ± 7.4	—
Hip circumference (cm)	105.3 ± 7.9	104.3 ± 6.2	—
Glucose (mg/dL)	88.7 ± 12.8	91.2 ± 31	74–109
GOT (U/L)	18.0 ± 6.9	22.6 ± 19.9	8–39
GPT (U/L)	23.8 ± 17.4	27.8 ± 19.1	<41
BUN (mg/dL)	13.5 ± 3.8	12.3 ± 3.2	7–25
Creatinine (mg/dL)	0.70 ± 0.16	0.71 ± 0.17	0.6–1.3
Triglyceride (mg/dL)	111.1 ± 58.7	122.3 ± 87.4	<150
Total cholesterol (mg/dL)	197.0 ± 39.2	186.4 ± 43.7	<200
HDL-C (mg/dL)	54.3 ± 12.9	53.0 ± 11.2	≥40 (M), ≥50 (F)
LDL-C (mg/dL)	125.5 ± 34.9	112.5 ± 31.9	<130

Data are presented as mean ± SD or n (%). Abbreviations: BMI, body mass index; GOT, glutamate-oxaloacetate transaminase; GPT, glutamate-pyruvate transaminase; BUN, blood urea nitrogen; HDL-C, high-density lipoprotein cholesterol; LDL-C, low-density lipoprotein cholesterol.

Supplementary Table S2. Safety assessment of the AKK formula in the post-randomization available-case population

Parameter	AKK formula (n = 33)	Placebo (n = 34)	Reference range
**Liver function**			
GOT (U/L)	18.0 ± 6.9 → 19.2 ± 6.7	22.6 ± 19.9 → 21.5 ± 10.8	8 – 39
GPT (U/L)	23.8 ± 17.4 → 28.5 ± 28.3	27.8 ± 19.1 → 31.2 ± 32.2	< 41
Total bilirubin (mg/dL)	0.6 ± 0.2 → 0.6 ± 0.3	0.7 ± 0.3 → 0.7 ± 0.2	0.3 – 1.0
**Kidney function**			
BUN (mg/dL)	13.5 ± 3.8 → 13.7 ± 3.6	12.3 ± 3.2 → 13.7 ± 3.0	7 – 25
Creatinine (mg/dL)	0.70 ± 0.16 → 0.72 ± 0.17	0.71 ± 0.17 → 0.76 ± 0.15	0.6 – 1.3
Uric acid (mg/dL)	5.8 ± 1.4 → 5.6 ± 1.6	5.4 ± 1.5 → 5.5 ± 1.3	M: 4.4 – 7.6 / F: 2.3 – 6.6
**Thyroid and metabolism**			
TSH (μIU/mL)	2.2 ± 1.2 → 2.1 ± 1.3	2.0 ± 1.0 → 2.1 ± 1.0	0.38 – 5.33
**Hematology**			
RBC ($\times10^6$/μL)	4.8 ± 0.4 → 4.8 ± 0.4	4.9 ± 0.4 → 4.9 ± 0.5	M: 4.5–6.0 / F: 4.0–5.2
Hemoglobin (g/dL)	13.8 ± 1.8 → 13.8 ± 1.8	14.0 ± 1.4 → 14.3 ± 1.5	M: 13.5–17.5 / F: 11.5–15.5
Platelet count ($\times10^3$/μL)	264 ± 65 → 261 ± 73	281 ± 78 → 290 ± 86	130 – 400

Data are presented as mean ± SD. No clinically relevant adverse changes were observed after 8 weeks of supplementation. Abbreviations: GOT, glutamate-oxaloacetate transaminase; GPT, glutamate-pyruvate transaminase; BUN, blood urea nitrogen; TSH, thyroid-stimulating hormone; RBC, red blood cell.

Supplementary Table S3. Daily dietary intake during the intervention period

Variable	Pre	Post-4	Post-8
Calories (kcal)			
AKK	1609.28±422.63	1523.40±482.00	1534.20±387.76
Placebo	1457.96±356.23	1583.63±407.14	1590.08±425.36
Carbohydrate (g)			
AKK	164.72±45.91	163.31±58.62	160.45±43.23
Placebo	156.65±45.48	170.27±48.30	158.91±57.82
Protein (g)			
AKK	76.65±23.50	72.32±25.65	72.62±20.53
Placebo	69.29±18.97	71.51±19.68	73.09±21.51
Fat (g)			
AKK	67.56±26.84	60.72±24.18	64.44±19.60
Placebo	57.88±17.14	64.60±19.94*	69.70±20.02*
Fiber (g)			
AKK	5.02±2.66$^{\#}$	3.78±2.84	3.58±2.82*
Placebo	3.60±1.82	2.80±1.87	3.22±2.81

Data are presented as mean ± SD. Pre, baseline; Post-4, week 4; Post-8, week 8. AKK group, n = 25; placebo group, n = 24. * $p < 0.05$ compared with Pre; # $p < 0.05$ compared with the placebo group.

Supplementary Table S4. Pairwise PERMANOVA results based on Bray–Curtis dissimilarities

Contrast			R^2	p	p (FDR)
AKK formula Week 8	vs	Placebo Week 8	0.012	0.699	0.855
Placebo Week 0	vs	Placebo Week 8	0.011	0.791	0.855
AKK formula Week 0	vs	AKK Formula Week 8	0.010	0.855	0.855
AKK formula Week 0	vs	Placebo Week 0	0.013	0.593	0.855
AKK formula Week 8	vs	Placebo Week 0	**0.023**	**0.074**	**0.444**
AKK formula Week 0	vs	Placebo Week 8	0.017	0.287	0.855

R^2 represents the proportion of variance explained. P-values were adjusted using the false discovery rate (FDR) procedure.

Supplementary Table S5. Summary of PICRUSt2 prediction quality and MetaCyc pathway analysis

Metric	Value
Median (IQR) NSTI	0.064 (0.048–0.077)
Mean (range) NSTI	0.066 (0.030–0.140)
MetaCyc pathway tests (Week 0, Week 8)	No pathways passed $q < 0.05$ (FDR)

NSTI, Nearest Sequenced Taxon Index; IQR, interquartile range; FDR, false discovery rate.

Supplementary Table S6. Post-randomization available-case analysis of qPCR outcomes in participants with follow-up data (n = 67)

Characteristics	AKK formula (n = 33)		Placebo (n = 34)	
	Week 0	Week 8	Week 0	Week 8
Relative abundance of *A. muciniphila*	1.0 fold	0.33 fold	1.0 fold	0.61 fold
The proportion of subjects who experienced an increase in *A. muciniphila* of more than 10%	-	75.8%#	-	36.4%

# $p < 0.05$ compared with the placebo group. Statistical analysis for responder proportion was performed using Fisher's exact test, with a ≥10% increase in *A. muciniphila* set as the threshold. Note: This supplementary analysis was performed in the post-randomization available-case population (n = 67) as a robustness assessment. The main qPCR analysis reported in Table 4 was conducted in the per-protocol population (n = 50).

Supplementary Table S7. Effects of the AKK formula on anthropometric measurements in the post-randomization available-case population

Characteristics	AKK formula (n = 33)					Placebo (n = 34)						
	Week 0	Week 4	Week 8	Δ_{4-0} (mean, 95% CI)	Δ_{8-0} (mean, 95% CI)	Week 0	Week 4	Week 8	Between-group Δ_{4-0} (mean diff, 95% CI)	Between-group Δ_{8-0} (mean diff, 95% CI)	p-value (Δ_{8-0})	p-value (Δ_{8-0}) (FDR-adjusted)
Weight (kg)	80.26±14.77	80.21±14.76	80.04±14.74	-0.05 (-0.43, 0.32)	-0.22 (-0.78, 0.35)	77.58±10.89	77.42±10.88	77.6±11.01	0.10 (-0.40, 0.61)	-0.25 (-0.96, 0.48)	0.5017	0.7296
BMI (kg/m^2)	28.44±3.01	28.43±3.04	28.37±3.10	-0.02 (-0.15, 0.12)	-0.07 (-0.27, 0.13)	28.18±3.19	28.13±3.21	28.19±3.21	0.04 (-0.14, 0.22)	-0.08 (-0.33, 0.17)	0.5306	0.7296
Waist (cm)	93.70±9.13	94.12±9.78	93.60±9.96	0.42 (-0.73, 1.58)	-0.1 (-1.31, 1.11)	91.99±7.43	92.57±7.19	91.88±7.17	-0.16 (-1.70, 1.37)	0.005 (-1.71, 1.72)	0.9945	0.9945
Hip (cm)	105.27±7.91	104.18±7.32*	104.18±7.40*	-1.09 (-2.07, -0.11)	-1.09 (-2.06, -0.12)	104.28±6.21	102.78±5.88	103.04±5.89*	0.41 (-1.41, 2.23)	0.14 (-1.17, 1.46)	0.8266	0.9093
WHR (%)	0.890±0.055	0.904±0.055*	0.898±0.060	0.014 (0.001, 0.027)	0.008 (-0.004, 0.020)	0.883±0.045	0.902±0.058*	0.893±0.047	0 (-0.02, 0.01)	0 (-0.02, 0.01)	0.8149	0.9093

Data are presented as mean ± SD. Δ_{4-0} and Δ_{8-0} indicate the changes from week 0 to week 4 and from week 0 to week 8, respectively. P-values for between-group comparisons were adjusted using the false discovery rate (FDR) procedure. Abbreviations: BMI, body mass index; WHR, waist-to-hip ratio; CI, confidence interval.

Supplementary Table S8. Effects of the AKK formula on blood lipids and metabolic variables in the post-randomization available-case population

Characteristics	AKK formula (n = 33)			Placebo (n = 34)				
	Week 0	Week 8	Δ_{8-0} (mean, 95% CI)	Week 0	Week 8	Between-group Δ_{8-0} (mean diff, 95% CI)	p-value (Δ_{8-0})	p-value (Δ_{8-0}) (FDR-adjusted)
Total cholesterol (mg/dL)	197.00±39.20	195.06±44.89	-1.94 (-10.15, 6.27)	186.38±43.74	195.12±36.56	-10.68 (-23.31, 1.96)	0.0964	0.4777
LDL-C (mg/dL)	125.48±34.89	124.33±38.48	-1.15 (-7.88, 5.58)	112.47±31.93	123.68±34.07*	-12.36 (-21.08, -3.63)	0.0062	0.0682
HDL-C (mg/dL)	54.28±12.90	55.93 ± 14.17	1.65 (-0.23, 3.53)	53.00±11.21	55.62±10.15*	-0.96 (-3.76, 1.83)	0.4942	0.7296
Glucose (mg/dL)	88.67±12.83	90.42±11.65	1.76 (-0.21, 3.73)	91.24±30.95	91.06±32.35	1.93 (-1.40, 5.27)	0.2504	0.5509
Insulin (μU/mL)	16.32±10.91	14.72±7.70	-1.60 (-4.26, 1.06)	14.25±8.89	15.35±8.63	-2.70 (-6.37, 0.98)	0.1474	0.4777
Insulin resistance	3.64±2.56	3.33±1.91	-0.31 (-0.89, 0.27)	3.34±2.91	3.59±2.92	-0.56 (-1.38, 0.25)	0.1737	0.4777

Data are presented as mean ± SD. Δ_{8-0} indicates the change from week 0 to week 8. * $p < 0.05$ compared with week 0 (baseline). P-values for between-group comparisons were adjusted using the false discovery rate (FDR) procedure. Abbreviations: HDL-C, high-density lipoprotein cholesterol; LDL-C, low-density lipoprotein cholesterol; CI, confidence interval.

Supplementary Table S9. Gastrointestinal symptom rating scale scores in the post-randomization available-case population

Characteristics	AKK formula (n = 33)					Placebo (n = 34)						
	Week 0	Week 4	Week 8	Δ_{4-0} (mean, 95% CI)	Δ_{8-0} (mean, 95% CI)	Week 0	Week 4	Week 8	Between-group Δ_{4-0} (mean diff, 95% CI)	Between-group Δ_{8-0} (mean diff, 95% CI)	p-value (Δ_{8-0})	p-value (Δ_{8-0}) (FDR-adjusted)
Total GSRS score	24.1 ± 6.7	24.2 ± 9.5	22.7 ± 5.4	0.09 (-2.52, 2.70)	-1.39 (-2.91, 0.12)	25.7 ± 8.2	25.1 ± 6.4	25.5 ± 7.9	0.71 (-2.71, 4.13)	-1.16 (-3.91, 1.59)	0.4018	0.7992
Abdominal pain	3.9 ± 1.1	4.1 ± 2.0	3.7 ± 1.0	0.21 (-0.42, 0.84)	-0.15 (-0.55, 0.25)	4.1 ± 1.6	3.8 ± 1.3	4.1 ± 1.5	0.45 (-0.40, 1.30)	-0.15 (-0.79, 0.49)	0.6386	0.7992
Reflux	2.5 ± 1.5	2.4 ± 0.8	2.3 ± 0.7	-0.09 (-0.63, 0.44)	-0.15 (-0.54, 0.24)	2.5 ± 1.1	2.4 ± 0.8	2.5 ± 0.8	-0.003 (-0.63, 0.63)	-0.15 (-0.71, 0.40)	0.5879	0.7992
Indigestion	8.1 ± 3.3	8.4 ± 4.3	8.2 ± 3.2	0.24 (-0.86, 1.35)	0.03 (-0.76, 0.82)	9.3 ± 3.6	9.5 ± 3.2	9.1 ± 3.4	0.10 (-1.35, 1.54)	0.30 (-1.03, 1.62)	0.6582	0.7992
Diarrhea	5.1 ± 2.2	5.3 ± 3.5	4.5 ± 1.6	0.21 (-0.67, 1.09)	-0.55 (-1.13, 0.04)	5.1 ± 2.6	4.9 ± 2.3	4.9 ± 2.2	0.45 (-0.78, 1.68)	-0.40 (-1.30, 0.50)	0.3793	0.7992
Constipation	4.6 ± 1.6	4.1 ± 1.4	4.0 ± 0.9*	-0.48 (-1.01, 0.04)	-0.58 (-1.14, -0.01)	4.7 ± 2.2	4.5 ± 1.9	4.9 ± 2.4	-0.28 (-1.12, 0.56)	-0.75 (-1.64, 0.14)	0.0953	0.5463

Data are presented as mean ± SD. Δ_{4-0} and Δ_{8-0} indicate the changes from week 0 to week 4 and from week 0 to week 8, respectively. * $p < 0.05$ compared with week 0 (baseline). *P*-values for between-group comparisons were adjusted using the false discovery rate (FDR) procedure. Abbreviations: GSRS, Gastrointestinal Symptom Rating Scale; CI, confidence interval.
